# Differential Cargo Mobilisation within Weibel-Palade Bodies after Transient Fusion with the Plasma Membrane

**DOI:** 10.1371/journal.pone.0108093

**Published:** 2014-09-18

**Authors:** Nikolai I. Kiskin, Victor Babich, Laura Knipe, Matthew J. Hannah, Tom Carter

**Affiliations:** 1 Division of Physical Biochemistry, Medical Research Council National Institute for Medical Research, London, United Kingdom; 2 Division of Neurophysiology, Medical Research Council National Institute for Medical Research, London, United Kingdom; 3 Department of Medicine, University of Maryland School of Medicine, Baltimore, Maryland, United States of America; 4 Microbiology Services Colindale, Public Health England, London, United Kingdom; 5 Cardiovascular and Cell Sciences Research Institute, St George’s University, London, United Kingdom; Institut Curie, France

## Abstract

Inflammatory chemokines can be selectively released from Weibel-Palade bodies (WPBs) during kiss-and-run exocytosis. Such selectivity may arise from molecular size filtering by the fusion pore, however differential intra-WPB cargo re-mobilisation following fusion-induced structural changes within the WPB may also contribute to this process. To determine whether WPB cargo molecules are differentially re-mobilised, we applied FRAP to residual post-fusion WPB structures formed after transient exocytosis in which some or all of the fluorescent cargo was retained. Transient fusion resulted in WPB collapse from a rod to a spheroid shape accompanied by substantial swelling (>2 times by surface area) and membrane mixing between the WPB and plasma membranes. Post-fusion WPBs supported cumulative WPB exocytosis. To quantify diffusion inside rounded organelles we developed a method of FRAP analysis based on image moments. FRAP analysis showed that von Willebrand factor-EGFP (VWF-EGFP) and the VWF-propolypeptide-EGFP (Pro-EGFP) were immobile in post-fusion WPBs. Because Eotaxin-3-EGFP and ssEGFP (small soluble cargo proteins) were largely depleted from post-fusion WPBs, we studied these molecules in cells preincubated in the weak base NH_4_Cl which caused WPB alkalinisation and rounding similar to that produced by plasma membrane fusion. In these cells we found a dramatic increase in mobilities of Eotaxin-3-EGFP and ssEGFP that exceeded the resolution of our method (∼2.4 µm^2^/s mean). In contrast, the membrane mobilities of EGFP-CD63 and EGFP-Rab27A in post-fusion WPBs were unchanged, while P-selectin-EGFP acquired mobility. Our data suggest that selective re-mobilisation of chemokines during transient fusion contributes to selective chemokine secretion during transient WPB exocytosis. Selective secretion provides a mechanism to regulate intravascular inflammatory processes with reduced risk of thrombosis.

## Introduction

WPBs are endothelial cell-specific regulated secretory granules containing polymers of VWF condensed into ordered helical tubules through a non-covalent association with the cleaved VWF-propolypeptide (Pro-VWF, Pro-) [1]–[3]. VWF secreted from WPBs plays an important role in haemostasis, but under pathophysiological conditions may contribute to thrombosis [4]. In addition to VWF, WPBs may contain a cocktail of pro-inflammatory molecules including P-selectin, IL-8, IL-6, monocyte chemoattractant protein 1 (MCP-1), growth-regulated oncogene-α (GROα) or Eotaxin-3 [5], [6]. Because secretion of these molecules from WPBs may play a role in inflammatory processes within the vasculature, it is important to understand how such molecules gain entry to these organelles [6]–[8], how they are stored [9], mobilized and released during exocytosis.

Imaging studies show that WPBs can release either all or selected components of their cargo [10]. Selectivity of release is thought to result from formation of a narrow fusion pore that acts as a molecular size filter allowing exit of low molecular weight cargo (e.g. chemokines, 5-HT) and ions (e.g. H^+^) [10], [11]. Consistent with this, combined amperometric and optical analysis of WPB fusion has shown standalone foot signals of large amplitude coincident with fusion events associated with selective release [11]. Similar modes of granule fusion occur in other secretory cell types [12]–[14], suggesting that control of fusion pore formation and size represents one important mechanism for selective cargo release for granules containing complex mixtures of molecules. It is suggested that post-fusion re-mobilisation of organelle cargo is a crucial step in cargo release [15]–[17]. Because soluble WPB cargo proteins and P-selectin are stored in the mature organelle in an immobile or very slowly mobile state [9], the extent to which such cargo is re-mobilised within the WPB after plasma membrane fusion may determine the availability for diffusive release. To address this we applied quantitative confocal FRAP to measure protein mobilities within individual structures formed after secretagogue-evoked transient fusion of WPBs with the plasma membrane. For comparison, mobilities in spherical WPBs produced after preincubation in the weak base NH_4_Cl [9] were also studied. The mobilities of WPB cargo on the surface of spherical membrane-bounded structures (membrane proteins) were analysed by previously published methods [9], [23]–[25]. To analyse mobilities of cargo diffusing inside WPBs (soluble proteins), we developed extended mathematical methods for FRAP analysis.

We found that post-fusion WPBs swell substantially and estimated that the mobilities of small soluble molecules (Eotaxin-3-EGFP, ssEGFP) increased by more than two orders of magnitude. In contrast, larger soluble proteins (VWF, Pro-VWF and tissue plasminogen activator (tPA)) remained immobile. The differential changes in cargo mobility determined here favour selective release of inflammatory molecules during transient modes of exocytosis, allowing control of inflammation with a reduced risk of thrombosis.

## Materials and Methods

### Cell culture and reagents

Human umbilical vein endothelial cells (HUVECs) were purchased, cultured, nucleofected and transferred to Rose chambers with glass bottom (#1.0, 0.15 mm, VWR International, UK) coated with collagen or 35-mm diameter glass-bottomed dishes (MatTeK Corp.), either directly or after preincubation in NH_4_Cl for 3 hours at 37°C for complete WPB rounding, as previously described [9]. VWF-EGFP, Pro-EGFP, Pro-mRFP, tPA-EGFP, P-selectin-EGFP, EGFP-Rab27A, ssEGFP, EGFP-CD63, EGFP-Rab35 and Eotaxin-3-EGFP constructs were made or obtained as previously described [9], [10], [18]–[21]. Alexa Fluor 647 Hydrazide (Alexa-647) and Vibrant DiI (vDiI) were purchased from Invitrogen. All other reagents were from Sigma-Aldrich. For studies with EGFP-CD63, cells were routinely co-transfected with Pro-mRFP, to ensure the selection of WPB-originating rounded structures. In some experiments, Pro-mRFP was not co-transfected with the membrane proteins P-selectin-EGFP or EGFP-Rab27A, as these membrane proteins uniquely identified WPBs. Post-fusion WPBs were identified soon after stimulation by their spheroid shape, the intra-luminal accumulation of the extracellular fluid phase marker Alexa-647, and the presence of fluorescent cargo.

### Imaging of WPB exocytosis

HUVEC cultures were placed on the confocal microscope stage in physiological saline [9] (in mM): NaCl- 140, KCl- 5, MgCl_2_- 1, CaCl_2_- 2, Glucose- 10, HEPES- 20, pH 7.3 (adjusted with NaOH), supplemented with 4–7 µM Alexa-647. Cells were stimulated with histamine (100 µM) or ionomycin (1 µM) at room temperature and imaged at 3–50 min after stimulation. Excitation laser lines were 488 nm (EGFP), 561 nm (mRFP, vDiI) and 633 nm (Alexa-647). While identifying the post-fusion objects, minimal emission windows were set to 500–530 nm, 585–625 nm and 700–790 nm, correspondingly. FRAP was performed on Leica Microsystems TCS SP2 or on higher speed Leica TCS SP5 confocal system (8 kHz resonant scanner), as previously described [9], [10], [22] using single-wavelength or sequential excitation (EGFP emission window 495–620 nm) with Leica HCX PL APO CS×100 oil-immersion objectives with 1.40 (SP2) or 1.46 (SP5) numerical apertures. Images of 512×128 pixels were collected at maximal zoom; for higher speed FRAP the number of horizontal scan lines was reduced to 64. Epifluorescence imaging of stimulated secretion of WPBs labelled with membrane proteins EGFP-CD63 or P-selectin-EGFP was done in physiological saline on a Deltavision system (Applied Precision Inc.), as previously described [20]. Unless otherwise indicated, all experiments were conducted at room temperature (21–23°C).

### FRAP in post-fusion or NH_4_Cl-rounded WPBs

Post-fusion or NH_4_Cl-rounded structures were bleached using a region of interest (ROI) partially (∼20–60%) covering the area with 1–2 scans at maximal power in bidirectional (“fly”) mode, and subsequent fluorescence recovery was recorded with highest possible speed (maximum rate 18 ms per frame) in the first seconds of recovery. The thickness of HUVEC in the cell periphery did not exceed 1–2 µm, so the confocal pinhole was opened wildly to record whole object fluorescence. To verify that intracellular post-fusion structures were isolated, at the end of FRAP experiment we completely bleached the structure to register the loss of signal. Protein diffusion on the surface of spherical WPB structures was quantified by the method of normalised first moment of membrane fluorescence [23]–[25], based on co-integration of experimental data profile with Legendre polynomial of the first order 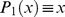, i.e. with the ramp function. This method allowed quantification of the lateral diffusion (coefficient *D*) on the surface of a sphere of radius *R*_0_, based on the time constant of the single-exponential decay *τ* of the normalised first image moment which represents the time constant of recovery:
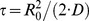
(1)


To analyse diffusion *inside* a spherical body, we extended this approach.

### The method of moments (normal-mode analysis) for quantitative treatment of diffusion inside the spherical body

**Figure 1:**
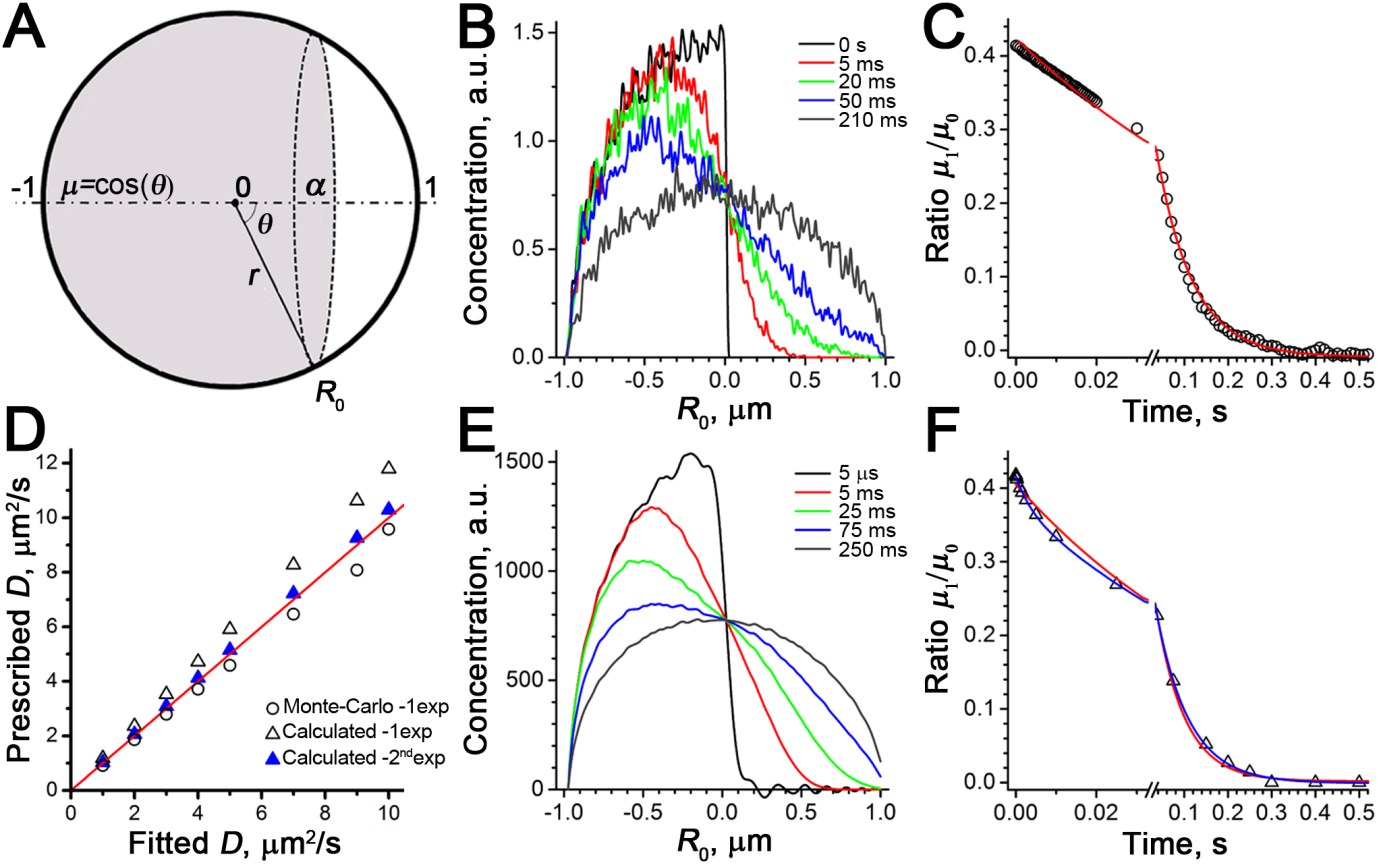
Modelling post-bleaching diffusion inside a sphere: Monte-Carlo simulations and direct calculations. *A*, Graphical representation of the bleaching experiment, showing an impermeable sphere of unit radius, in which the horizontal polar axis distance *µ* = cos(*θ*) and the normalised radius *r* are spherical coordinates describing fluorophore concentration distribution. A bleached region covering the horizontal axis interval *α*≤*µ*≤1 is shown in white, the rest of the sphere contains fluorophore (grey). *B–C* Monte-Carlo FRAP recovery simulations. *B,* Concentration profiles for simulated redistribution of 20000 particles, initially positioned at time *τ* = 0 s in the left hemisphere (black trace). Recovery times are coded by the trace colours. Simulation parameters were: sphere radius *R*_0_ = 1 µm, prescribed diffusion coefficient *D* = 3 µm^2^/s, bleaching parameter *α* = 0, simulation step 5·10^−4 ^s. Profiles were constructed from images representing the 2D projections of the sphere, each simulated particle was represented by a pixel of value 1. The plot of normalised moment kinetics is shown in *C*. Red line- monoexponential fit, the fitted time constant *τ* = 83 ms corresponds to the *D* value of 2.79 µm^2^/s (Eq.8). *E–F,* Analysis of the same problem from images representing projections of concentration distributions calculated using Eq.10. *E*, Concentration profiles for simulated distributions, time-dependent colour coding as in *B*. Oscillating patterns (Gibb’s phenomena) at short times (black trace) are due to approximation of discontinuity by finite Legendre sums. *F,* The normalised moment kinetics fitted either by a single exponent (red trace, *τ* = 66 ms, evaluated *D* = 3.5 µm^2^/s) or by a sum of two exponents (blue trace, *τ*_1_ = 3.5 ms, *τ*_2_ = 75 ms, *D* evaluated from *τ*_2_ was 3.1 µm^2^/s; the amplitude of fast component was 0.105 of that for slow component). *D,* The relationship between the values of *D* prescribed in Monte-Carlo simulations and results obtained from fits as in *C* and *F.* The results were compared with identity line (red). The slopes of the best fits were: for Monte-Carlo simulations (as in *B–C*) 0.93±0.009 (circles), for the best monoexponential fit to calculated values 1.18±0.0006 (open triangles), for the slower mode of bi-exponential fit (as in *E–F*) 1.03±0.0004 (filled blue triangles).



We describe diffusion inside a sphere of radius *R*_0_ partially filled with a substance of initial concentration *C*_0_ using normalised values of concentration *c* = *C*/*C*_0_, of radius *r* = *R*/*R*_0_ and of time *τ* = *D·t*/*R*_0_^2^. An unbleached part of the sphere (grey region in Fig. 1A) lies beyond a line based on polar angle 0<*θ*_0_<π, for any azimuthal angle 0<*φ*<2π in vertical section planes. This region contains fluorescent protein, which diffuses into the rest of the sphere. In normalised coordinates (*r*, *µ*, *τ*), where *µ* = cos(*θ*) (horizontal axis in Fig. 1A), we obtain the differential equation for diffusion of unbleached fluorophore.

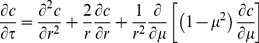
(2)


with the initial post-bleach condition of all the fluorescence contained in the area -1≤*µ*<cos(*θ*_0_) = *α* (initial distribution, see grey-shaded area Fig. 1A):



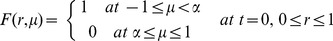
(3)


and with boundary condition for impermeable sphere



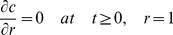
(4)The value of *α* in this context represents the bleaching parameter, varying from 1 (no bleaching) through 0 (half-bleaching) up to −1 (complete bleaching). The Eqs.2–4 can be solved by the method of separation of variables. In the most general form, the complete solution of this equation [26] is.
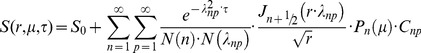
(5)where 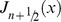 is the Bessel function of the first kind and (*n*+1/2) order, 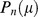 are Legendre polynomials of order *n*, the mean average concentration in the sphere *S*_0_ and coefficients *C*_np_ are defined by the initial fluorophore distribution *F*_0_(*r*,*µ*) (Eq.3):
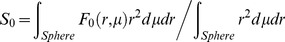


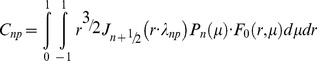

*N*(*n*) and *N*(*λ*_np_) are the norms of eigenfunctions (Legendre polynomials and Bessel functions correspondingly) and *λ*_np_ are the roots of the equation:



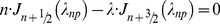
(6)In analogy with the diffusion problem on the surface of a sphere [23], we may multiply the solution Eq.5 by the 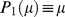 and integrate the expression by *r* and by *µ* on the whole sphere volume to obtain the first moment of distribution *µ*_1_:
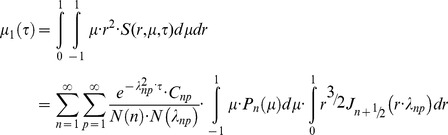



Using the orthogonality properties of Legendre polynomials, we find that the above expression is non-zero only when *n* = 1, and thus the moment is expressed as an infinite sum of exponents.
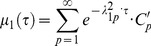
(7)


From Eq.6 solved numerically for *n* = 1, the roots *λ* ^2^_1p_ in Eq.7 form an increasing sequence (4.333, 35.288, 84.748, …). Thus, sequential time constants of exponential terms in the Eq.7 decrease steeply, and we may consider the first term as a main slowest mode. On return to the time variable, we find that the main component of diffusion-dependent moment kinetics *µ*_1_(*t*) is proportional to
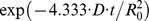



defining the time constant of fluorescence recovery
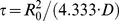
(8)


This expression was used for quantitation of mobilities for proteins diffusing *inside* the rounded organelles, while the Eq.1 was used for membrane diffusion confined to the *surface* of the organelle.

### Data analysis

The minimal constant fluorescence count outside the object of study was considered as background. In background-subtracted images the polygon ROI was set to enclose the whole rounded structure. Using a custom-made analysis macro implemented in ImageJ (http://rsb.info.nih.gov/ij/), the ROI position was adjusted frame-by-frame either automatically or manually to maximise the mean fluorescence values and thus follow the structure outline and account for minor object jitter. For each time frame, the image moments *µ*_0_ (average) and *µ*_1_ (1^st^ moment) were calculated from image profiles by pixel value summation (Fig. 1B) representing mean intensities of vertical pixel columns in round ROI plotted over horizontal distance (*µ* after normalisation, Fig. 1A). The time course of decline of the normalised moment *µ*_1_/*µ*_0_ with fluorescence recovery was fitted by decaying exponent. The peak value of *µ*_1_/*µ*_0_ reflected the maximal asymmetry of the image immediately after bleaching (*α*), the diffusion coefficient *D* was found from the time constant of decay *τ* using Eq.1 or Eq.8, for surface or bulk diffusion, correspondingly and the steady-state value represented the residual asymmetry, i.e. the immobile fraction [25]. Correspondingly, the mobile fraction (MF) was defined by subtracting this value from 100%. The radius of structure *R*_0_ was estimated in ImageJ by fitting an ellipsoid to the enclosing ROI and taking the mean of its half-axes. For doughnut-like round structures formed with membrane protein markers, *R*_0_ was determined from the position of fluorescence maxima in the central xy-section. An upper limit for the reliable quantitation of *D* (the critical *D* value, *D*_crit_) for each experiment was chosen to be.
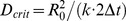
(9)


with *k* equal to 2 or 4.333 for diffusion on the surface or inside the spherical body correspondingly, where Δ*t* - an interval between image acquisition frames at the fastest phase of recovery. The results showing *D*>*D*_crit_ (2 points or less to fit exponent) were discarded as unresolved.

All results were expressed as mean±s.e.m. Statistical significance P values in nonparametric statistical comparisons of means between independent groups were calculated using two-tailed Mann-Whitney test, for ANOVA Kruskal-Wallis test was performed with data pairs compared in follow-up Dunn’s multiple comparisons tests. The critical level of statistical significance was P<0.05. All statistical calculations were performed in GraphPad Prism 5.02 or 6.

### Verifications of the model by Monte-Carlo simulations and numerical calculations

To model diffusion within a sphere after half sphere bleaching (*θ*_0_ = π/2, *α* = 0), we randomly positioned 20000 particles in the left half of a sphere radius *R*_0_ = 1 µm and allowed them to “diffuse” into the entire volume. In 0.5–0.05 ms time intervals each particle was allowed to jump the distance randomly selected from the isotropic 3D-normal distribution based on a prescribed diffusion coefficient *D*. If a particle was about to cross the sphere boundary, it was reflected back from the point of surface crossing. Snapshot projected images of the particles’ distribution in the sphere were created using custom ImageJ plugins, collected at user-defined time points and analysed using the same ImageJ macro as for experimental FRAP data. We found (Fig. 1B–C) that for prescribed values of *D* in the range 1–10 µm^2^/s, model diffusive re-equilibration occurred on a sub-second time scale, and the fits to the kinetics of the measured moment change looked invariably monoexponential (Fig. 1C). All plotting and exponential fitting were done in OriginLab Origin 8.5.1.

Alternatively, we verified the conclusions of the model Eqs.3–5 by analysing the model images of recovery representing projections of scaled theoretical concentration distributions *C*(*r, µ, t*) for the same model conditions as above. For integer values of *n* from 1 to 36 we evaluated numerically (PTC Mathcad 14) 12 sequential roots of the Eq.6 building the 36×12 matrix *λ*_np_. By putting *α* = 0 into Eq.5, simplifying and evaluating *C*_np_ we obtain
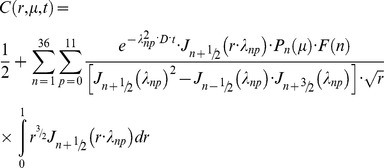



with
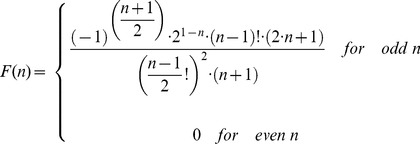
(10)


Furthermore, because we record the total fluorescence from the sphere volume (open pinhole), we accounted for this in images of the sphere in Cartesian coordinates by multiplying the concentration/fluorescence in each point (*x*, *y*) by the number of points factor 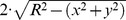 (or 2*R*_0_⋅sin(*θ*) in spherical coordinates). From an array of such images based on Eq.10 for different time points, we obtained similar plots of moment kinetics, again using the same processing and fitting as for experimental data (Fig. 1 E–F). As expected, the calculations produced the results similar to Monte-Carlo simulations (Fig. 1 B–C), except that kinetics of the moment Fig. 1F appeared bi-exponential. A time constant of the faster mode was 21 times faster than that of the main mode, but accounted just for 0.1 of the main mode amplitude (mean for all 8 calculations, Fig. 1F).

Fig. 1D demonstrates a good correspondence between the prescribed *D* (ordinate) and the result evaluated by measurements in Monte-Carlo simulations (circles) or model analysis from Eq.10 (triangles). In the latter case, the slope of the best fit was closest to unity for the slower mode of the bi-exponential fit, while the single-exponential fit overestimated *D* values.

## Results

### WPB exocytosis: shape change, swelling and membrane mixing

**Figure 2:**
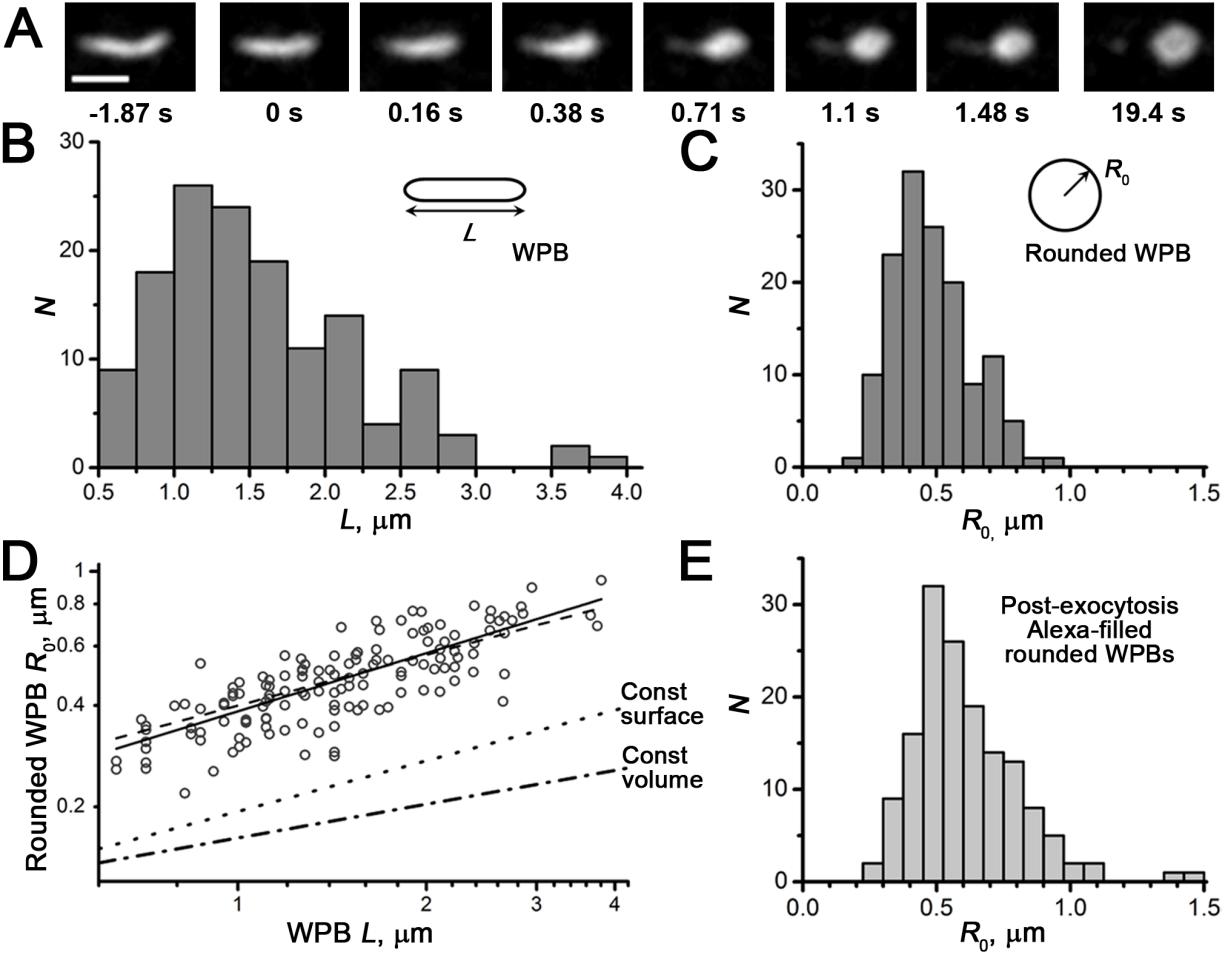
Post-fusion WPBs swell. *A*, Example of the WPB shape change seen during stimulated (1 µM ionomycin) fusion in a HUVEC expressing EGFP-CD63. Scale bar, 2 µm. *B–C*, The distributions of the pre-fusion WPB lengths *L* (*B,* mean 1.56±0.06 µm) and of maximal radii of the resulting spheroid structures *R*_0_ seen soon after fusion (*C,* mean 0.49±0.01 µm) for all WPBs measured when undergoing exocytosis (labelled with EGFP-CD63, *n* = 99 and with P-selectin-EGFP, *n = *41). *D,* The radius of rounded structure (*C*) plotted against the corresponding length of the parent WPB (*B*) in the same fusion event (points), double logarithmic scale. The solid line is the best power function fit to the dependence, *R*_0_ = 0.384·*L* ^0.573^, the dashed line is the best √*L* fit: *R*_0_ = 0.399·√*L*. The dotted line represents radius prediction for the model of rounding preserving the WPB surface, *R*_0_ = 0.194·√*L,* the dash-dot line represents the constant-volume rounding model, *R*_0_ = 0.162·*L* ^1/3^. In calculations the average diameter of WPBs was assumed to be 0.15 µm. *E*, the distribution of radii for all rounded fluorescent WPBs found persisting after stimulation and containing extracellular Alexa-647 (mean radius 0.61±0.02 µm, *n* = 150).



WPBs undergo a profound shape change during hormone-stimulated exocytosis (Fig. 2A, [10], [20]). Using epifluorescence time-lapse imaging, we followed the relationship between pre-fusion WPB length *L* (Fig. 2B) and the radius *R*_0_ of the rounded structure formed upon plasma membrane fusion of the same WPB (Fig. 2C). The dependence of the resulting *R*_0_ on the parent pre-fusion WPB length *L* (Fig. 2D) was fitted by the general power function 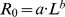 (solid line). The best power fit exponent b was 0.573, close to 0.5, suggesting a dependence on the √*L* (dashed line). Such dependence can potentially be a consequence of the spherical structure having the same surface as the parent WPB. Representing the WPB as a very thin cylinder of radius *r* = 0.075 µm (obtained from measurements of WPB diameters in EM images 2*r* = 150 nm, [1], [3], [27], [28]) the surface will be approximately 2π*rL* and the average radius of the same-area sphere can be calculated as

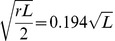
(in µm)This dependence is plotted in Fig. 2D as a dotted line (Constant surface) which lies well below the measured data. To account for the measured difference, the area of the WPB membrane would have to increase on rounding 0.399/0.194 = 2.06 times proportionally to the area of the parent WPB. The estimate of radius based on the preservation of the WPB volume (the Constant volume line in Fig. 2D) lies even further from the measured data. Confocal FRAP analysis was also carried out on rounded post-fusion WPBs found persisting inside the cell several minutes after stimulation and containing extracellular Alexa-647 dye inside. Pre-FRAP measurements of the radii of these post-fusion WPBs (mean 0.61±0.02 µm, *n* = 150, Fig. 2E) showed them to be slightly, but significantly (P<10^−4^), larger than those seen directly after fusion in epifluorescence experiments (0.49±0.012 µm, *n* = 140, Fig. 2C), indicating that after transient fusion further swelling might occurred.

**Figure 3:**
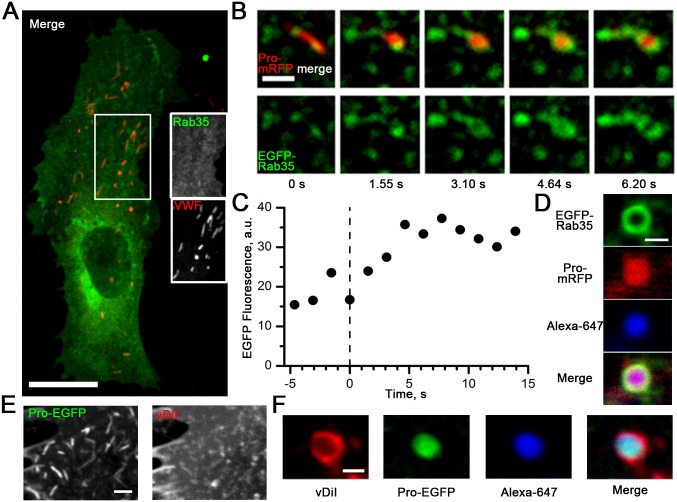
Post-fusion WPBs acquire plasma membrane components by membrane mixing. *A*, Image of a HUVEC expressing EGFP-Rab35 membrane protein (green) and immunolableled for endogenous VWF (red). Greyscale images are from the region indicated by the white box. Scale bar, 10 µm. *B*, Top, a montage of colour-merged images of a Pro-mRFP-labelled WPB (red) from a confocal live cell video of HUVEC co-expressing Pro-mRFP- and EGFP-Rab35- (green) and undergoing histamine-evoked exocytosis. *B*, Bottom, the EGFP-Rab35 component alone. Scale bar, 2 µm. The images show entry of plasma membrane EGFP-Rab35 into the membrane of the fusing WPB. *C*, The kinetics of EGFP-Rab35 fluorescence accumulation in the WPB shown in *B* (radius of the ROI used for measurements was 0.75 µm). The time constant of the exponential fit to the protein transfer process was 3.16 s. *D,* Confocal images of rounded WPB found inside HUVEC after being stimulated in the presence of the extracellular fluid phase marker Alexa-647. Plasma membrane-derived EGFP-Rab35 (green) is found in the limiting membrane of the post-fusion WPB structure containing Alexa-647 dye (blue) and positive for Pro-mRFP (red). Scale bar, 1 µm. Experiments *B*–*D* were performed at 37°C. *E–F,* Membrane dye vDiI transfers from plasma membrane to the post-fusion WPB. *E*, Right, confocal fluorescence of vDiI in a HUVEC containing Pro-EGFP-labelled WPBs (left image). Scale bar, 2 µm. *F*, Confocal images of a post-fusion WPB expressing Pro-EGFP (green) showing the presence of vDiI (red) after stimulation in the presence of the extracellular fluid phase marker Alexa-647 (blue). Scale bar, 1 µm.

The increased surface area of post-fusion WPBs assumes acquisition of lipid and possibly protein components from the plasma membrane. Membrane mixing during histamine-evoked WPB exocytosis was confirmed using cells co-expressing the plasma membrane-targeted Rab protein, EGFP-Rab35 [29] and Pro-mRFP (Fig. 3A). Dual-colour confocal imaging revealed a clear time-dependent accumulation of EGFP-Rab35 around WPBs as they rounded up after fusion (Fig. 3B–C). During fusion EGFP-Rab35 incorporated into the WPB membrane with a mean time constant of 1.82±0.51 s. The mean radius of the “doughnut-like” EGFP-Rab35-positive WPB structure 0.55±0.03 µm (*n* = 7) corresponded well to the size of rounded WPBs. Rab35 from plasma membrane was also detected in post-fusion WPB structures persisting inside the cell after stimulation (Fig. 3D). To investigate further the mixing of membrane components, we used the membrane dye vDiI in cells expressing Pro-EGFP. vDiI labelled the plasma membrane and some internal membranes, most likely due to endocytosis, but crucially not WPBs (Fig. 3E). After histamine stimulation, vDiI was found in the limiting membranes of all post-fusion spheroid WPBs (Fig. 3F). Together the data show that post-fusion WPBs swell and acquire plasma membrane-associated components by lateral diffusion.

### Post-fusion WPBs support cumulative WPB exocytosis

**Figure 4:**
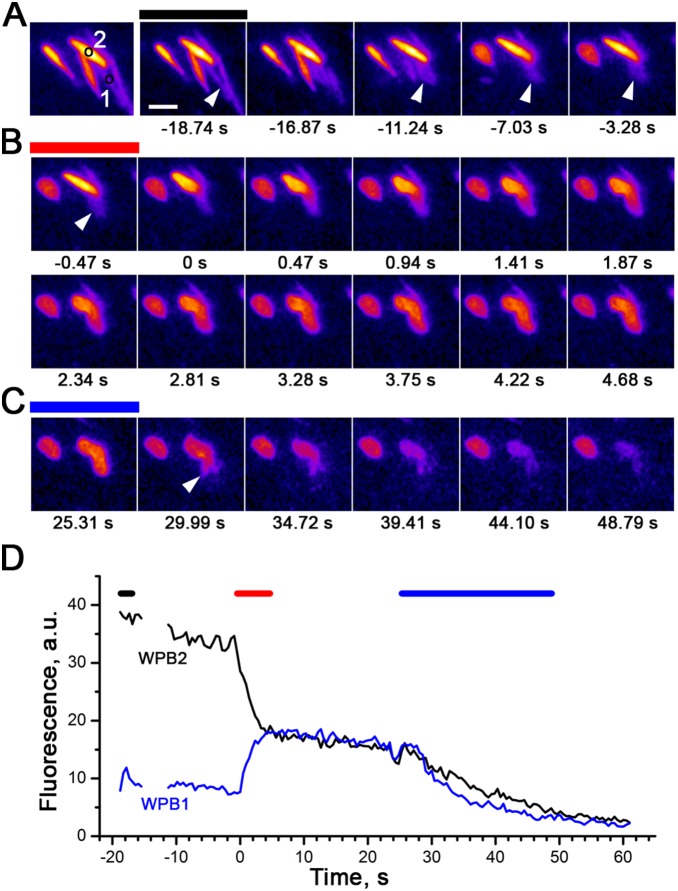
Cumulative fusion involving post-fusion WPB. Selected images from a live cell epifluorescence video (see also Movie S1) of an EGFP-CD63-expressing HUVEC stimulated with 100 µM histamine. Scale bar, 2 µm. *A*, Left, approximate positions of ROIs corresponding to two EGFP-CD63-labelled WPBs participating in the cumulative fusion. *A*, Right, images showing the change in shape of the first, dimmer WPB (ROI ‘1’) due to fusion. Arrow in frame 2 of *A* indicates a WPB that fuses to form initial rounded structure, indicated by arrows in frames 4–6 and frame 1 of *B*. Here and below the colour-coded bars above the images correspond to the marked time intervals in the graph *D*. *B*, Two rows of images showing the fusion of the second WPB ‘2’ with the rounded WPB ‘1’ indicated in *A.* The time of this second fusion was set to 0 s. Note the redistribution of fluorescence between the two structures. *C*, Loss of EGFP-CD63 fluorescence of the compound structure (arrow) upon fusion with the plasma membrane. *D*, The time course of the fluorescence changes within ROI ‘1’ (blue) and ROI ‘2’ (black) in control (black bar), during the cumulative fusion event (red bar) and after fusion with plasma membrane (blue bar). The average intensities of images were normalised to the beginning of experiment. The slow residual decline of fluorescence is due to uncompensated photobleaching of EGFP.

During live cell imaging experiments we found evidence of cumulative WPB exocytosis involving post-fusion WPBs. Cumulative exocytosis was detected in ∼30% of cells (14/50 cells) with a mean frequency of 1.3±0.7 events per cell (*n* = 18 events in 14 cells). Cumulative fusion was identified as the re-distribution of mobile EGFP-CD63 molecules between the limiting membranes of newly-fusing WPB and a post-fusion granule. Fig. 4 and Movie S1 show an example of such an event. During histamine (100 µM) stimulation a rod-shaped WPB (marked by ROI ‘1’ in the first image Fig. 4A and the arrow in first frames of Movie S1) undergoes an abrupt change of shape typical for a post-fusion WPB. The location of the post-fusion structure is indicated by arrows in Fig. 4A–B and by the second arrow in Movie S1. Images in Fig. 4B show the subsequent fusion of a second, bright fluorescent WPB (marked by ROI ‘2’ in the first image Fig. 4A) with the post-fusion WPB ‘1’. The fluorescence intensity traces in Fig. 4D show the re-equilibration of EGFP-CD63 fluorescence between WPBs ‘1’ and ‘2’. Approximately 30 s after formation of the compound structure (arrow in Fig. 4C and third arrow in Movie S1), it lost its content due to fusion with the plasma membrane, resulting in complete dispersal of EGFP-CD63 fluorescence.

### Mobility of soluble proteins in post-fusion or NH_4_Cl-rounded WPBs

**Figure 5:**
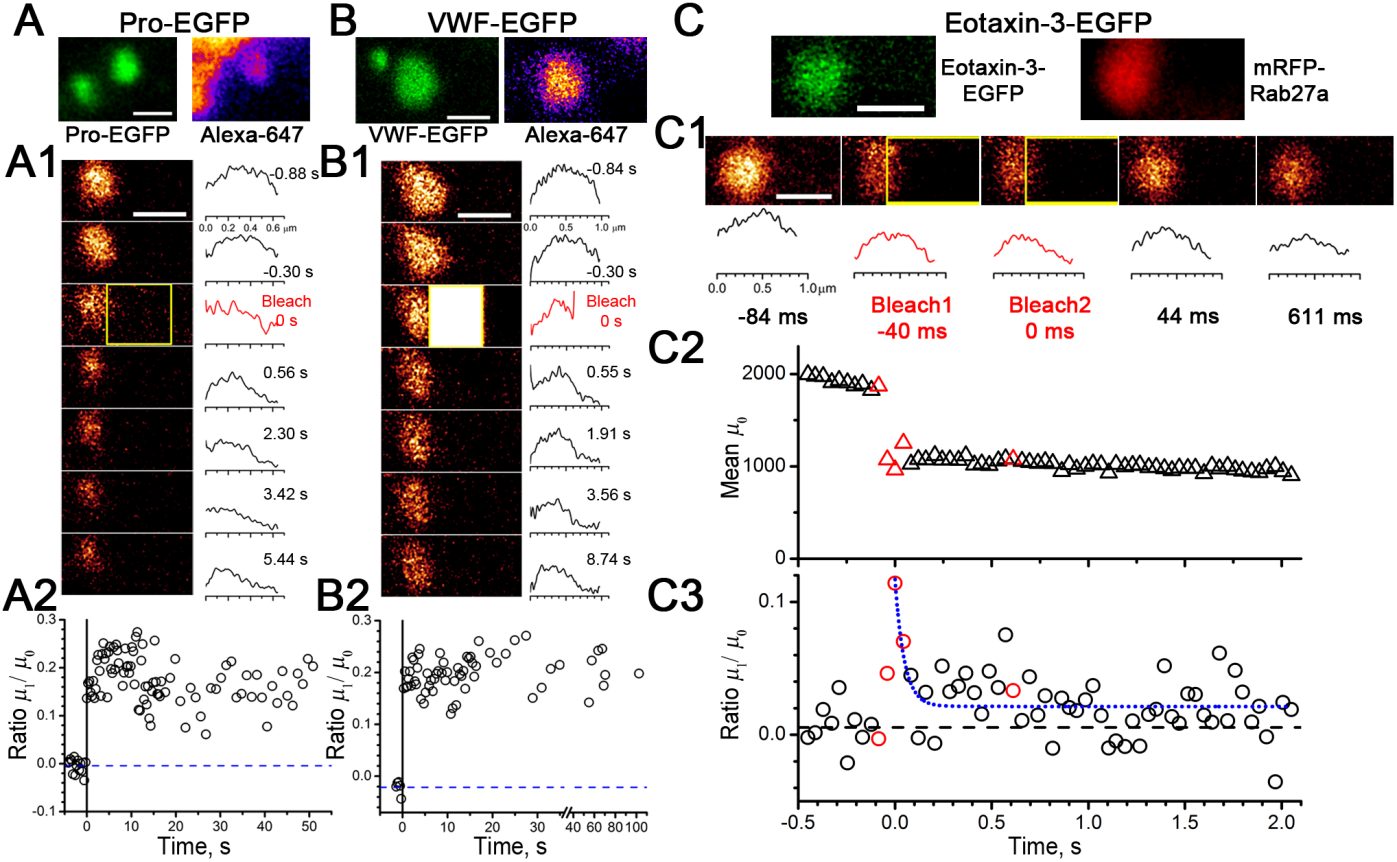
Differential changes in intra-WPB mobilities of soluble cargo proteins in post-fusion WPBs. *A–B*, Images of extracellular Alexa-647-containing (right panels) post-fusion WPBs expressing Pro-EGFP (*A*, green) or VWF-EGFP (*B*, green). *A1–B1*, Left, images and right, image profiles for selected FRAP times (as indicated) for a Pro-EGFP- (*A1*) or VWF-EGFP-containing (*B1*) post-fusion WPBs. Here and in subsequent images the bleaching frames are marked in yellow. *A2–B2*, The lack of recovery of normalised first moment for Pro-EGFP (*A2*) and VWF-EGFP (*B*2) indicating that both are immobile within the post-fusion structures. Scale bars, 1 µm. *C*, Image of an Eotaxin-3-EGFP- and mRFP-Rab27A-co-expressing WPB rounded by preincubation in NH_4_Cl. Both scale bars, 1 µm. *C1*, Top, images and bottom, fluorescence profiles for Eotaxin-3-EGFP fluorescence during a FRAP experiment. *C2*, The change of average fluorescence in the rounded structure (moment *µ*_0_). The symbols corresponding to the frames shown in the images *C1* above are coloured red. *C3*, The plot of the normalised first moment *µ*_1_/*µ*_0_ and an exponential fit to its kinetics (blue dotted line). The time constant of the fit, 48 ms, is unreliable as it is based only on 2–3 measured points. With mean frame interval 41 ms, *R*_0_ = 49 µm we estimate *D*_crit_ = 1.49 µm^2^/s, meaning the diffusion in this experiment is faster than this value.

To determine how intra-WPB cargo mobilities change after transient fusion, we carried out FRAP experiments in post-fusion WPBs containing Pro-EGFP (Fig. 5A) or VWF-EGFP (Fig. 5B). VWF-EGFP was immobile and, in most cases, so was Pro-EGFP (Table 1). However, in a small fraction of experiments (3/22) very slow recovery of Pro-EGFP was observed. Similar Pro-EGFP mobilities were previously found in NH_4_Cl-collapsed WPBs ([9] and Table 1). A similar immobility/slow mobility picture was seen for tPA-EGFP (Table 1). It proved difficult to find post-fusion WPBs retaining sufficient amounts of Eotaxin-3-EGFP or ssEGFP fluorescence for reliable FRAP analysis. Therefore, we assessed the NH_4_Cl-rounded WPBs, in which Eotaxin-3-EGFP or ssEGFP were fully retained. As Fig. 5C demonstrates, even with the fast time-resolution resonant scanner of the Leica TCS SP5, the kinetics of FRAP recovery with Eotaxin-3-EGFP was difficult to record. The non-normalised moment *µ*_0_ (average intensity, triangles in Fig. 5C2) clearly demonstrated a sharp drop, confirming the bleaching of internal fluorescence, however, the change in *µ*_1_/*µ*_0_ (circles in Fig. 5C3) was only noticeable in the first one-two post-bleaching frames. The high speed of recovery meant that *D* could not be reliably determined (*D*>*D*_crit_ = 0.69 µm^2^/s). For all *n* = 15 Eotaxin-3-EGFP FRAP experiments which demonstrated a clear decrease in average fluorescence, the *D*_crit_ was in the range 0.08–1.9 µm^2^/s (median 0.76 µm^2^/s), and in all cases the kinetics of recovery was too fast to quantify (*D*>*D*_crit_). In similar experiments with ssEGFP in all *n* = 13 experiments on NH_4_Cl-rounded WPBs, despite even higher resolution (*D*_crit_ range 0.34–4.39 µm^2^/s, median 1.42 µm^2^/s), we also obtained *D*>*D*_crit_. Assuming the mobility in post-fusion rounded WPBs is similar to that in NH_4_Cl-rounded WPBs, these data suggest that small soluble proteins become highly mobile, with the mobilities possibly exceeding the range reported for Eotaxin-3-EGFP/ssEGFP in the endoplasmic reticulum of HUVEC, 1–2 µm^2^/s [9].

**Table 1:** Comparison of mobilities of WPB proteins in NH_4_Cl-rounded and post-fusion WPBs.

WPB protein	a *n*_mob_/*n*_im_	*n*_total_		*D* b±s.e.m.	MF^b^±s.e.m.	*n*_MF_	*n*_mob_/*n*_im_	*n*_total_	*D*±s.e.m.	MF±s.e.m	*n*_MF_
	WPB µm^2^/s %	WPB µm^2^/s %
	WPBs rounded by prolonged exposure to NH_4_Cl						Post-secretion rounded WPBs containing Alexa-647				
VWF-EGFP	0/7	7	immobile		0		0/9	9	immobile	0	
Proregion-EGFP	3/9	12	0.0086±0.0025		19±10	12	3/19	22	0.0028±0.0024	11±6	22
			[0.018±0.0044]^c^		[76±12]	[3]			[0.020±0.0016]	[79±12]	[3]
tPA-EGFP	2/4	6	0.0016±0.012		26±17	6	0/5	5	immobile	0	
			[0.048±0.025]		[78±20]	[2]					
P-selectin-EGFP	13/0	13	0.063±0.021		92±2.2	12	16/0	16	0.077±0.011	84±3.1	14
EGFP-CD63	19/0	19	0.13±0.024		92±2.1	16	19/0	19	0.11±0.020	81±3.6	16
EGFP-Rab27A	14/0	14	0.90±0.18		95±1.7	20	9/0	9	0.69±0.18	97±1.7	9

a *n*_mob_ and *n*_im_ are numbers of WPB experiments where mobile or immobile behaviour was observed, correspondingly.

b *D* and MF (mobile fraction) values as calculated for all experiments, immobile values regarded as 0s.

c the values in square brackets single out mobile cases only.

### Mobility of membrane proteins in post-fusion and NH_4_Cl-rounded WPBs

**Figure 6:**
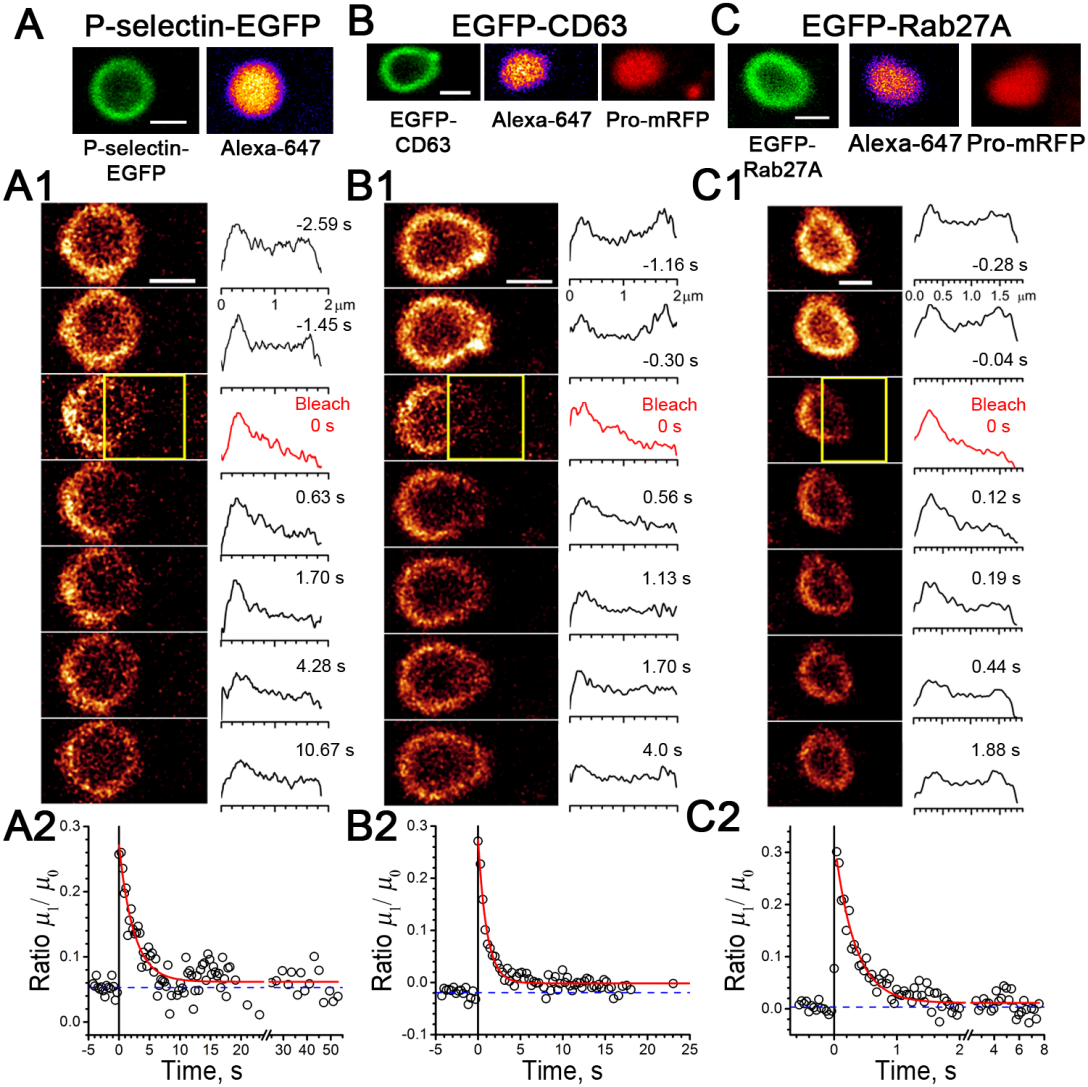
Mobilities of membrane proteins in post-exocytosis rounded WPBs. *A*, Image of a post-fusion P-selectin-EGFP-labelled WPB (left, green) containing the extracellular fluid phase marker Alexa–647 (right). *A1*, Left, images and right, image profiles measured at selected FRAP times (as indicated). *A2*, The time course of normalised moment kinetics fitted by exponent (red line), time constant *τ* = 2.44 s, with the radius *R*_0_ = 0.69 µm yielding the *D* value of 0.098 µm^2^/s (Eq. 1). *B*, Image of a post-fusion WPB co-expressing EGFP-CD63 (left, green) and Pro-mRFP (right, red) and labelled with the extracellular fluid phase marker Alexa-647 (middle). *B1*, Images and image profiles for EGFP-CD63 during FRAP. *B2*, Exponential fit to the normalised moment change, *τ* = 1.0 s, *R*_0_ = 0.78 µm, *D* = 0.29 µm^2^/s. *C,* Image of a post-fusion WPB co-expressing EGFP-Rab27A (left, green) and Pro-mRFP (right, red) and containing the extracellular fluid phase marker Alexa-647 (middle). *C1*, Images and image profiles for EGFP-Rab27A during FRAP. *C2*, Analysis of moment kinetics, exponential fit *τ* = 0.32 s, *R*_0_ = 0.62 µm, *D* = 0.58 µm^2^/s. All scale bars, 1 µm.

Fig. 6A–C summarises FRAP experiments in post-fusion WPBs containing fluorescently labelled membrane proteins. We have previously shown that P-selectin-EGFP was immobile in mature WPBs, but acquired mobility in NH_4_Cl-rounded WPBs ([9] and Table 1). Here we show that P-selectin-EGFP is re-mobilised within post-fusion rounded WPBs (Fig. 6A, Table 1), with a mobility not significantly different from that in NH_4_Cl-rounded structures (P = 0.21), but with a slightly reduced mobile fraction (P = 0.05). ANOVA comparison of *D* values measured for EGFP-CD63 and EGFP-Rab27A in post-fusion rounded (Fig. 6B–C), NH_4_Cl-rounded WPBs or mature WPBs [9], showed no significant differences (P = 0.78 EGFP-CD63, P = 0.87 EGFP-Rab27A), hence diffusional mobility of membrane proteins underwent no changes in exocytosis process. The mobile fraction for EGFP-CD63 was lower in post-fusion WPBs compared to NH_4_Cl-rounded organelles (P = 0.05), while for EGFP-Rab27A it was similar (P = 0.55).

## Discussion

### Transient WPB fusion: shape change, swelling and membrane mixing

WPB fusion proceeds via formation of an initially narrow pore connecting the organelle lumen and extracellular space [11]. At first, the WPB retains its rod-like shape, but after 30–200 ms it collapses to a spheroid [20] as the Pro-VWF and VWF tubules, which give the organelle its distinctive shape, become disordered [3]. In common with most other secretory granules [30]–[34], we found that WPBs rapidly swelled upon fusion (Fig. 2). The mechanisms underlying secretory granule swelling have been extensively discussed (see [34] and references therein); for WPBs they likely include an increase in repulsive forces between the polyanionic VWF and Pro-VWF macromolecules following loss of cationic shielding species (e.g. H^+^, Ca^2+^, amines etc., see [21] and references therein). The forces created by secretory granule swelling have not been studied in detail, but pressure estimates [33] show that they may alone be sufficient for the rapid and explosive expulsion of content, as seen for VWF release during hormone-evoked WPB exocytosis [19]. An active process of actomyosin assembly and contraction has been proposed to underlie VWF release from post-fusion WPBs formed during phorbol ester action [35]. However, the process of actomyosin assembly occurs on a time scale of many seconds after WPB fusion, too slow to account for the subsecond delays between WPB fusion and the explosive expulsion of VWF observed during high-speed imaging of WPB exocytosis under hormone or calcium ionophore action [20]. Indeed, WPB fusion into external media buffered to pH 6.5 prevents the fusion-evoked shape change and explosive expulsion of VWF, although small molecule release is largely unaffected [21]. This suggests that rapid VWF explusion is most likely driven by loss of cationic shielding species and water entry, similar to the mechanism shown to underlie rapid expulsion of mucin glycoproteins during mucin granule exocytosis (see [20] for detailed discussion and references).

We found that transient WPB fusion resulted in the accumulation of plasma membrane-associated Rab35 [29] or vDiI in the WPB membrane (Fig. 3). Fast entry of EGFP-Rab35 into the WPB membrane by lateral diffusion (Fig. 3B–D) indicates that the pore is likely to be largely lipidic in nature, consistent with the rapid loss of EGFP-CD63 from WPBs during “lingering kiss” fusions [10].

### Post-fusion WPBs support cumulative WPB fusion

Exchange of membrane components, including SNARE proteins [36], during transient fusion is suggested to render post-fusion granule membranes capable of supporting cumulative granule exocytosis [37]–[41], a process that may occur during stimulated WPB exocytosis [28]. Here we found direct evidence for cumulative WPB fusion at sites formed by post-fusion WPBs, although these events were rare (Fig. 4 and Movie S1). In lymphoid cells the physiological significance of cumulative fusion has been extensively discussed [42]. In other cell types it is proposed to direct granule contents toward specific sites determined by morphological characteristics [37], [38], [41]. In endothelial cells, compound or cumulative WPB fusion may concentrate VWF delivery at specific sites on the cell surface allowing formation of very long extracellular VWF strings that efficiently recruit platelets to the blood vessel walls at high shear [43].

### Differential changes in the intra-WPB mobility of soluble cargo after transient fusion

The relatively large size of post-fusion WPBs allowed us to quantify protein mobilities using FRAP not only on the surface, but also within the volume of spherical post-fusion structures due to a developed extended method of FRAP analysis based on the normalised moment approach [23], [24]. The method of moments was suggested earlier for quantitation of various diffusion problems [44]. The application of this method is simple, based on image analysis and exponential fitting, which, as simulations (Fig. 1D) show, can estimate the values of *D* with less than 10% error. An advantage of the extended method of FRAP analysis is that it is independent of the magnitude of bleaching parameter *α,* unlike the FRAP of mature WPBs and other finite small structures [9], [45]. In principle, using the approach described in [24], the theory of FRAP can be extended to the volume of ellipsoids of revolution.

In mature WPBs VWF and Pro-VWF are immobilised [9] through incorporation into helical tubules. WPB fusion disrupts the tubules [3], likely forming a complex mesh or gel of VWF polymers within the swollen post-fusion organelle. Within such a mesh/gel individual VWF polymer units may undergo a slow reptation, however such movements could not be resolved by our method. The immobility of VWF and Pro-VWF within the mesh/gel will favour retention of these proteins within the post-fusion granule, until expulsion of VWF during full release events [19].

Steric entrapment of small molecules within/between the condensed Pro-VWF/VWF tubules and/or a pH-dependent binding to VWF may account for the very low mobility of small molecules in mature WPBs [9]. Post-fusion WPBs contained very low levels of Eotaxin-3-EGFP or ssEGFP fluorescence, suggesting that these proteins were almost completely released during transient fusions. While this made post-fusion FRAP experiments impossible, analysis of NH_4_Cl-collapsed WPBs provided an estimate of the order of magnitude for the increase in small molecule mobility after WPB alkalinisation, hydration, swelling and disruption of Pro-VWF\VWF tubules. Based on estimates of the resolution limits of our experiments (mean *D*_crit_ 2.4 µm^2^/s; from Eq. 9 using 18 ms frame rate and mean radius of post-fusion rounded WPB 0.61 µm,), Eotaxin-3-EGFP mobility in post-fusion WPBs increased in relation to diffusion in mature WPBs by more than two orders of magnitude. If chemokines do bind VWF, as has been proposed [7], then an ion exchange mechanism, similar to that described for the release of serotonin from mast cell secretory granules [46], [47], release of adrenaline from chromaffin granules [48] or for secretion of acetylcholine from *Torpedo* synaptic vesicles [49], may operate to re-mobilise them from VWF core entrapment. Whether such an ion exchange process involves H^+^ flux remains unclear since acidification of the external media did not prevent chemokine secretion from WPBs [21].

The time for half-equilibration by diffusion within the sphere *τ*_e_ is described by the equation [50]:
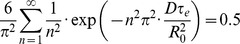



We may solve it obtaining 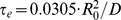. Substituting *D* = 1 µm^2^/s (most conservative slowest post-fusion estimate of small molecule mobility) and *R*_0_ = 0.49 µm, we obtain *τ*_e_ = 7.3 ms, much faster than the observed time course for small molecule release [10], [21]. As a result, within the rounded structure re-mobilised small proteins can be considered always in equilibrium. Thus, whatever the mechanism of small molecules’ re-mobilisation to high mobility, a consequence for WPB secretion is that their subsequent release will become independent of diffusion. In these conditions, the kinetics of their release into a large external volume will be exponential with time constant.
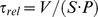



determined only by the organelle volume (*V*) to surface (*S*) ratio and pore permeability *P* [50]. For re-mobilised small proteins diffusing from the well-stirred sphere, the expression transforms to
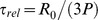



It follows that because of fast equilibrating diffusion on the size scale of post-fusion WPBs, for small proteins the time constant of their release will be governed by both structure dimensions and magnitude and kinetics of fusion pore permeability.

### Membrane protein mobilities in post-fusion WPBs

P-selectin is immobilised in the membrane of mature WPBs [9]. Here we show that P-selectin is re-mobilised after hormone-evoked transient plasma membrane fusion (Fig. 6A). The mechanisms underlying P-selectin immobilisation in WPBs have been discussed [9], and are likely to include steric entrapment of the large extracellular domain of the molecule within the condensed Pro-VWF/VWF tubules forming the core of the WPB. Tubule disruption after fusion will result in re-mobilisation of P-selectin. Both EGFP-CD63 and EGFP-Rab27A are mobile in the membrane of mature WPBs [9], and their mobilities remained unchanged in both hormone-evoked post-fusion WPBs and NH_4_Cl-collapsed WPBs. This suggests either that despite membrane mixing and exchange of components, the gross diffusive properties of the WPB membrane are not markedly altered after fusion or that these proteins are relatively insensitive to changes in the membrane environment. Alternatively, subtle changes in the modes of mobility could be undetectable by macroscopic FRAP methods.

## Conclusion

We have shown that transient WPB fusion selectively re-mobilises small WPB cargo. The high mobility of small proteins renders their release independent of diffusion, suggesting that the kinetics and extent of exocytosis is determined by the properties and regulation of the fusion pore connecting the WPB to the extracellular space. Immobility of pro-VWF/VWF favours retention of these proteins within post-fusion WPBs, making selective release of small molecules more probable. Thus, post-fusion changes within the WPB, in conjunction with fusion pore regulation will determine which molecules are faster available for release. The ability to selectively and quickly release small inflammatory chemokines prior to bulk WPB cargo may provide a mechanism to control inflammatory processes, while retention and delay of VWF/Pro-VWF release [10] reduces a risk of associated thrombosis.

## Supporting Information

Movie S1 **Cumulative WPB fusion.** Movie from which data in Figure 4 was derived. An EGFP-CD63-expressing HUVEC was stimulated with 100 µM histamine. First arrow points to a EGFPCD63-expressing WPB that undergoes transient fusion to form a post-fusion structure (indicated by second arrow). A second WPB fuses with the post-fusion structure at time 0 s resulting in redistribution of mobile EGFP-CD63 within the compound structure. Third arrow indicates approximate region where the compound structure fuses with the plasma membrane releasing EGFP-CD63. Scale bar, 2 µm.
(AVI)
